# Iron Deposition in Gray Matter Nuclei of Patients With Intracranial Artery Stenosis: A Quantitative Susceptibility Mapping Study

**DOI:** 10.3389/fneur.2021.785822

**Published:** 2022-01-05

**Authors:** Huimin Mao, Weiqiang Dou, Xinyi Wang, Kunjian Chen, Xinyu Wang, Yu Guo, Chao Zhang

**Affiliations:** ^1^Department of Radiology, The First Affiliated Hospital of Shandong First Medical University & Shandong Provincial Qianfoshan Hospital, Jinan, China; ^2^Postgraduate Department, Shandong First Medical University, Jinan, China; ^3^MR Research, GE Healthcare, Beijing, China

**Keywords:** anterior circulation artery stenosis, posterior circulation artery stenosis, ischemic stroke, quantitative susceptibility mapping, iron deposition, gray matter nuclei

## Abstract

**Purpose:** This study aimed to use quantitative susceptibility mapping (QSM) to systematically investigate the changes of iron content in gray matter (GM) nuclei in patients with long-term anterior circulation artery stenosis (ACAS) and posterior circulation artery stenosis (PCAS).

**Methods:** Twenty-five ACAS patients, 25 PCAS patients, and 25 age- and sex-matched healthy controls underwent QSM examination. Patients were scored using the National Institutes of Health Stroke Scale (NIHSS) and modified Rankin Scale (mRS) to assess the degree of neural function deficiency. On QSM images, iron related susceptibility of GM nuclei, including bilateral caudate nucleus, putamen (PU), globus pallidus (GP), thalamus (TH), substantia nigra (SN), red nucleus, and dentate nucleus (DN), were assessed. Susceptibility was compared between bilateral GM nuclei in healthy controls, ACAS patients, and PCAS patients. Partial correlation analysis, with age as a covariate, was separately performed to assess the relationships of susceptibility with NIHSS and mRS scores.

**Results:** There were no significant differences between the susceptibilities for left and right hemispheres in all seven GM nucleus subregions for healthy controls, ACAS patients, and PCAS patients. Compared with healthy controls, mean susceptibility of bilateral PU, GP, and SN in ACAS patients and of bilateral PU, GP, SN, and DN in PCAS patients were significantly increased (all *P* < 0.05). In addition, mean susceptibility of bilateral TH and SN in PCAS patients was significantly higher than in ACAS patients (both *P* < 0.05). With partial correlation analysis, mean susceptibility at bilateral PU of ACAS patients was significantly correlated with mRS score (*r* = 0.415, *P* < 0.05), and at bilateral PU in PCAS patients was correlated with NIHSS score (*r* = 0.424, *P* < 0.05).

**Conclusion:** Our findings indicated that abnormal iron metabolism may present in different subregions of GM nuclei after long-term ACAS and PCAS. In addition, iron content of PU in patients with ACAS and PCAS was correlated with neurological deficit scores. Therefore, iron quantification measured by QSM susceptibility may provide a new insight to understand the pathological mechanism of ischemic stroke caused by ACAS and PCAS.

## Introduction

Intracranial artery stenosis (ICAS) is one of the main causes of ischemic stroke, accounting for 30~50% of total ischemic stroke cases (1). In cerebral arterial system, ICAS can be classified into anterior circulation artery stenosis (ACAS) and posterior circulation artery stenosis (PCAS). For ACAS or PCAS, brain tissues in blood supply areas can become ischemic and hypoxic, promoting a series of pathophysiological reactions, such as neuronal hyperexcitability, mitochondrial death, free radicals release, apoptosis, necrosis, autophagy, and inflammation, to cause neuronal damage (2). Eventually, a range of brain dysfunction diseases, such as speech or motor disorders, cognitive dysfunction and emotional disorder will appear, causing great harm and heavy burden to patients, families, and society. Therefore, the repair of neural function after ischemic stroke has become a research focus in recent years.

Iron content quantification is essential to evaluate the level of normal neurophysiological functions (3). As a co-factor for many enzymes, iron is involved in various important physiological and biochemical processes in the brain, including the synthesis of DNA and protein, oxygen transport, electron transport, oxidative phosphorylation, myelinization, and the synthesis of neurotransmitters such as dopamine (4). However, iron overload caused by iron homeostasis imbalance can produce reactive oxygen species and oxidative stress to cause neuronal damage (5). Excessive iron deposition in brain has been identified in many neurological disorders, including cerebrovascular diseases, Parkinson's disease (PD), Alzheimer's disease (AD), and so on (6, 7). In pre-clinical ischemic stroke models caused by unilateral middle cerebral artery (MCA) occlusion, increased iron was reported to deposit in the lesioned hemisphere, and iron deposition could exacerbate neuronal damage during ischemia/reperfusion (8). When ICAS occurs, cerebral cells of the corresponding blood supply region, including vascular endothelial cells, can become ischemic and hypoxic, which in turn leads to the destruction of blood-brain barrier. Endothelial cells are key regulators of iron transport, and blood-brain barrier is an important structure for regulating iron transport and metabolism in the brain (9, 10). When both structures are damaged, iron circulation homeostasis is altered and excessive iron deposits in the brain. Inadequate blood flow and oxygen supply induced by ICAS can also trigger a cascade of pathological non-infectious neuroinflammation, leading to impaired iron homeostasis in the central nervous system (11). In addition, previous studies have shown that iron chelation can attenuate ischemia/reperfusion damage in animal models (12, 13). Therefore, iron deposition may be a potential biomarker for ICAS.

Due to the invasive nature of pathological examination for iron quantification, many iron related neurological studies only stayed at pre-clinical phase. Consequently, a reliable non-invasive method for quantitative iron assessment *in vivo* has been ideally required. Quantitative susceptibility mapping (QSM), a promising and non-invasive magnetic resonance imaging (MRI) technique, can measure susceptibility difference between magnetic tissues *in vivo* based on magnetic gradient echo MR phase data (14). QSM can be used to evaluate iron content in brain, particularly in gray matter (GM) nuclei, where iron is a strong paramagnetic source that increases the magnetic susceptibility of a tissue (15). Previous studies have demonstrated that susceptibility measured on QSM data was positively correlated with chemically determined iron concentration in brain tissue (16, 17). Moreover, QSM has been used to identify iron metabolism disorders for many neurological diseases such as AD, PD, and Vascular Dementia (18–20). Du et al. (21) used QSM to investigate alterations of iron content in bilateral basal ganglia of brain for patients with MCA occlusion, and found that iron-related average susceptibility in bilateral putamen (PU) of patients was significantly increased. While MCA stenosis, belonging to ACAS, is the most common ICAS subtype selected to investigate the pathophysiological mechanism of ischemic stroke, the mortality and disability rates of PCAS-related ischemic stroke are much higher than those of ACAS (22). However, PCAS has received little attention so far, especially concerning the changes of iron metabolism in ischemic stroke secondary to PCAS.

Abnormal iron metabolism can aggravate neurological damage through oxidative stress in a vicious cycle (5). The National Institutes of Health Stroke Scale (NIHSS) and the modified Rankin Scale (mRS) scores are the most commonly used clinical scales to evaluate the degree of neural function deficiency in ischemic stroke (23). Higher NIHSS or mRS scores indicate more severe neurological deficit (23). Sun et al. (24) found that iron related QSM susceptibility of thalamus (TH) in cerebral autosomal dominant arteriopathy with subcortical infarcts and leukoencephalopathy (CADASIL) patients was positively correlated with mRS score, indicating that excessive iron deposition may exacerbate clinical symptoms of patients with CADASIL. However, few studies have quantitatively evaluated brain iron deposition in both ACAS and PCAS patients and its correlation with neurological function.

Therefore, the main goal of this study was to systematically investigate the potential changes of iron content in bilateral GM nuclei in patients with long-term ACAS and PCAS using QSM technique, and to explore its correlations with neurological deficit scores.

## Materials and Methods

### Subjects

After obtaining Medical Ethics Committee of First Affiliated Hospital of Shandong First Medical University approval and informed consent, 50 ischemic stroke patients diagnosed with long-term ICAS and 25 healthy controls were recruited from November 2019 to September 2021. All enrolled patients were confirmed *via* clinical symptoms, conventional MRI examination, magnetic resonance angiography (MRA), or digital subtraction angiography. All 50 patients were subdivided into ACAS group (*n* = 25, mean age 56.88 ± 10.93 years, 15 males, and 10 females), including vascular stenosis of internal carotid artery (intracranial segment), MCA, anterior cerebral artery, and PCAS group (*n* = 25, mean age 56.76 ± 10.47 years, 18 males, and 7 females), including vascular stenosis of posterior cerebral artery (PCA), basilar artery, vertebral artery (intracranial segment). At admission, all patients underwent clinical evaluations, using NIHSS and mRS scores to assess the degree of neural function deficiency (23). Inclusion criteria of patients were defined as follows: (1) only ACAS or PCAS involved; (2) long-term stroke symptoms associated with offending arteries stenosis; and (3) no previous history of other confounding nervous system diseases, such as cerebral hemorrhage, subarachnoid hemorrhage, brain tumor, brain injury, PD, AD, and dementia. The exclusion criteria of patients included (1) concurrence of ACAS and PCAS related ischemic stroke; (2) stroke associated with cardiac embolism; (3) contraindications to MR examination; (4) incomplete clinical data; and (5) severe artifacts on MRI images.

In addition, 25 healthy controls (mean age 56.80 ± 10.73 years, 14 males, and 11 females) were also recruited in this study. Each subject had no cerebrovascular disease, brain injury, neurological, psychiatric, metabolic, or other systemic diseases that may affect the nervous system. All 25 healthy controls were confirmed without ICAS by MRA and without obvious abnormalities or only small lacunar infarcts by routine MRI.

### Imaging Acquisition

All MRI experiments were performed on a 3.0 T MRI (Discovery MR750, GE Healthcare, USA) equipped with a 32-channel phase-array head coil. All participants underwent routine MRI, including T1-weighted imaging, fast-spin-echo based T2-weighted imaging, T2 fluid-attenuated inversion recovery, and diffusion-weighted imaging, to exclude other cerebral organic diseases. Three-dimensional time-of-flight MRA was acquired to confirm the location of affected intracranial artery in patients and the absence of ICAS in healthy controls.

Three-dimensional spoiled gradient echo was used for QSM imaging (first TE = 3.0 ms, TE interval = 3.1 ms, number of TEs = 8, TR = 28.1 ms, FOV = 240 × 240 mm, flip angle = 20°, matrix size = 240 × 240, bandwidth = 62.50 kHz, slice thickness = 2 mm, number of slices = 64, NEX = 0.7, scanning time = 2 min 31 seconds, ASSET (Array Spatial Sensitivity Coding Technique) with acceleration factor of 2 was used for the acceleration method).

### Imaging Analysis

Several post-processing steps were performed to generate QSM maps using STI Suite embedded in MATLAB R2017b (MathWorks, Natick, MA) (25). Firstly, with a Laplacian-based method, unwrapped phase images were created from wrapped phase images (25). Secondly, the brain mask was generated from magnitude images using the brain extraction tool (BET) in FSL (FMRIB, University of Oxford, Oxford, UK) (26). Thirdly, the sophisticated harmonic artifact reduction for phase data with varying spherical kernel sizes (V-SHARP) method, with the spherical kernel radius ranging from 1 to 12 mm, was employed to remove background fields (25). Finally, QSM images were obtained by using the least-squares (LSQR)-algorithm-based method to calculate dipole inversion (25, 27).

**Figure 1:**
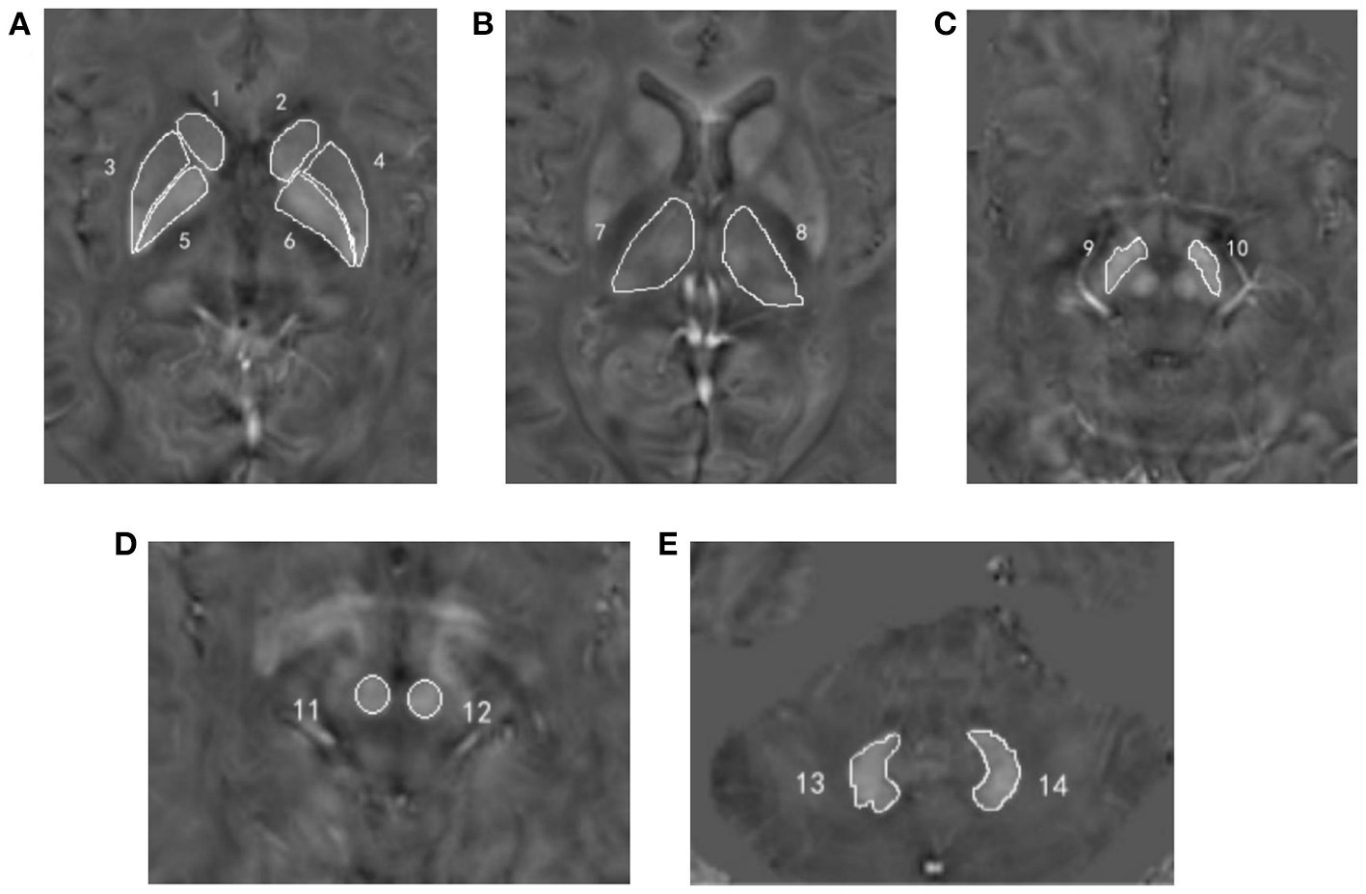
Regions of interest depicted on the quantitative susceptibility mapping images. **(A)** Number 1, 2 = bilateral caudate nucleus; number 3, 4 = bilateral putamen; number 5, 6 = bilateral globus pallidus; **(B)** number 7, 8 = bilateral thalamus; **(C)** number 9, 10 = bilateral substantia nigra; **(D)** number 11, 12 = bilateral red nucleus; **(E)** number 13, 14 = bilateral dentate nucleus.

The software ImageJ 1.52 (National Institutes of Health, Bethesda, MD, USA) was used to quantitatively measure the susceptibility values of each region of interest (ROI) on QSM images, including bilateral caudate nucleus (CN), PU, globus pallidus (GP), TH, substantia nigra (SN), red nucleus (RN), and dentate nucleus (DN) (Figure 1). All ROIs were manually drawn on three continuous slices that could clearly show the boundary of GM nucleus by two radiologists (XYW and HMM) with more than 3 years of experience in diagnosing central nervous system. Both observers were blinded to clinical and imaging information of all subjects. Mean susceptibility values for each ROI over two radiologists were recorded.

### Statistical Analysis

All statistical analyses were performed using GraphPad Prism 8.0 (GraphPad Software, Inc., La Jolla, CA, USA) and IBM SPSS 22.0 (Armonk, NY, USA). The Kolmogorov–Smirnov test was used to analyze the normality of continuous variables. Continuous variables with normal distribution were expressed as the mean ± standard deviation. Non-normally distributed continuous variables were represented as median and interquartile range (IQR). Counting data were represented as frequency and percentage (%). To compare the demographic and clinical data among healthy controls, ACAS, and PCAS patients, one-way analysis of variance was used for age, and Mann–Whitney *U*-test was used to analyze continuous variables with non-normal distribution. The chi-square (χ^2^) test was used for counting data and *Z*-test was used for the *post hoc* analysis, adjusted by the Bonferroni correction. Disease duration between ACAS and PCAS patients was assessed by independent sample *t*-test. The inter-observer consistency of susceptibility measurements was evaluated by intra-class correction coefficient (ICC). The resultant ICC > 0.75 was considered as good reproducibility. Paired *t*-test was used to compare the susceptibility between the left and right GM nuclei in healthy controls, ACAS, and PCAS patients, respectively. Independent sample *t*-test was used to separately compare the susceptibility differences between each two of healthy controls, ACAS patients and PCAS patients. Partial correlation analysis, with age as a covariate, was performed to assess the relationships of the susceptibility levels with NIHSS and mRS scores. Statistically significant threshold was set as *P* < 0.05.

## Results

### Demographic and Clinical Characteristics

Twenty-five patients with ACAS, 25 patients with PCAS, and 25 age- and sex-matched healthy controls were included in this study. These three groups showed no significant difference in age and sex (*P* = 0.999; *P* = 0.477, Table 1). Among patients with ACAS, the median (IQR) NIHSS score was 5 points (2–7), and the mRS score was 2 points (1–3). Among patients with PCAS, the median (IQR) NIHSS score was 3 points (2–7), and the mRS score was 1 point (1–2). Detailed clinical characteristics of all subjects were summarized in Table 1.

**Table 1:** Demographic and clinical characteristics of all participants.

	**Healthy controls (*n* = 25)**	**Patients with ACAS (*n* = 25)**	**Patients with PCAS (*n* = 25)**	**Statistical value**	***P-*value**
Age (years)	56.80 ± 10.73	56.88 ± 10.93	56.76 ± 10.47	0.001	0.999
Sex (male)	14 (56.0%)	15 (60.0%)	18 (72.0%)	1.482	0.477
Disease duration (years)	–	4.78 ± 2.71	6.02 ± 3.88	−1.310	0.197
NIHSS score [median (IQR)]	–	5 (2–7)	3 (2–7)	−0.589	0.556
mRS score [median (IQR)]	–	2 (1–3)	1 (1–2)	−1.543	0.123
Hypertension	3 (12.0%)	16 (64.0%)	18 (72.0%)	21.230	0.000^*ab^
Diabetes	2 (8.0%)	12 (48.0%)	10 (40.0%)	10.294	0.006^*ab^
Hyperlipidemia	–	8 (32.0%)	7 (28.0%)	0.095	0.758
Hyperhomocysteinemia	–	6 (24.0%)	7 (28.0%)	0.104	0.747
History of smoking	4 (16.0%)	10 (40.0%)	12 (48.0%)	6.122	0.047^*ab^
History of drinking	3 (12.0%)	8 (32.0%)	14 (56.0%)	10.920	0.004^*b^

*ACAS, anterior circulation artery stenosis; PCAS, posterior circulation artery stenosis; NIHSS, National Institutes of Health Stroke Scale; mRS, modified Rankin scale; IQR, interquartile range*.

* *P < 0.05. Z-test was used for the post hoc analysis, adjusted by the Bonferroni correction*:

a *Significant differences between healthy controls and patients with ACAS*.

b *Significant differences between healthy controls and patients with PCAS*.

### Inter-Observer Agreement Analysis

ICC analysis showed high inter-observer agreements for susceptibility measurements in all seven GM nucleus subregions between both radiologists (0.850 ≤ ICCs ≤ 0.960, Supplementary Table 1).

### Susceptibility Comparisons Between Left and Right GM Nucleus Subregions in Healthy Controls, Patients With ACAS, and Patients With PCAS

Paired *t*-test showed no significant differences between the left and right susceptibility in all seven GM nucleus subregions for healthy controls, patients with ACAS, and patients with PCAS, respectively (all *P* > 0.05, Supplementary Table 2). Therefore, the mean susceptibility values of each bilateral GM nuclei subregions were used for healthy controls, ACAS patient and PCAS patient groups in further data analyses, respectively.

### Susceptibility Comparisons Across Healthy Controls, Patients With ACAS, and Patients With PCAS

**Figure 2:**
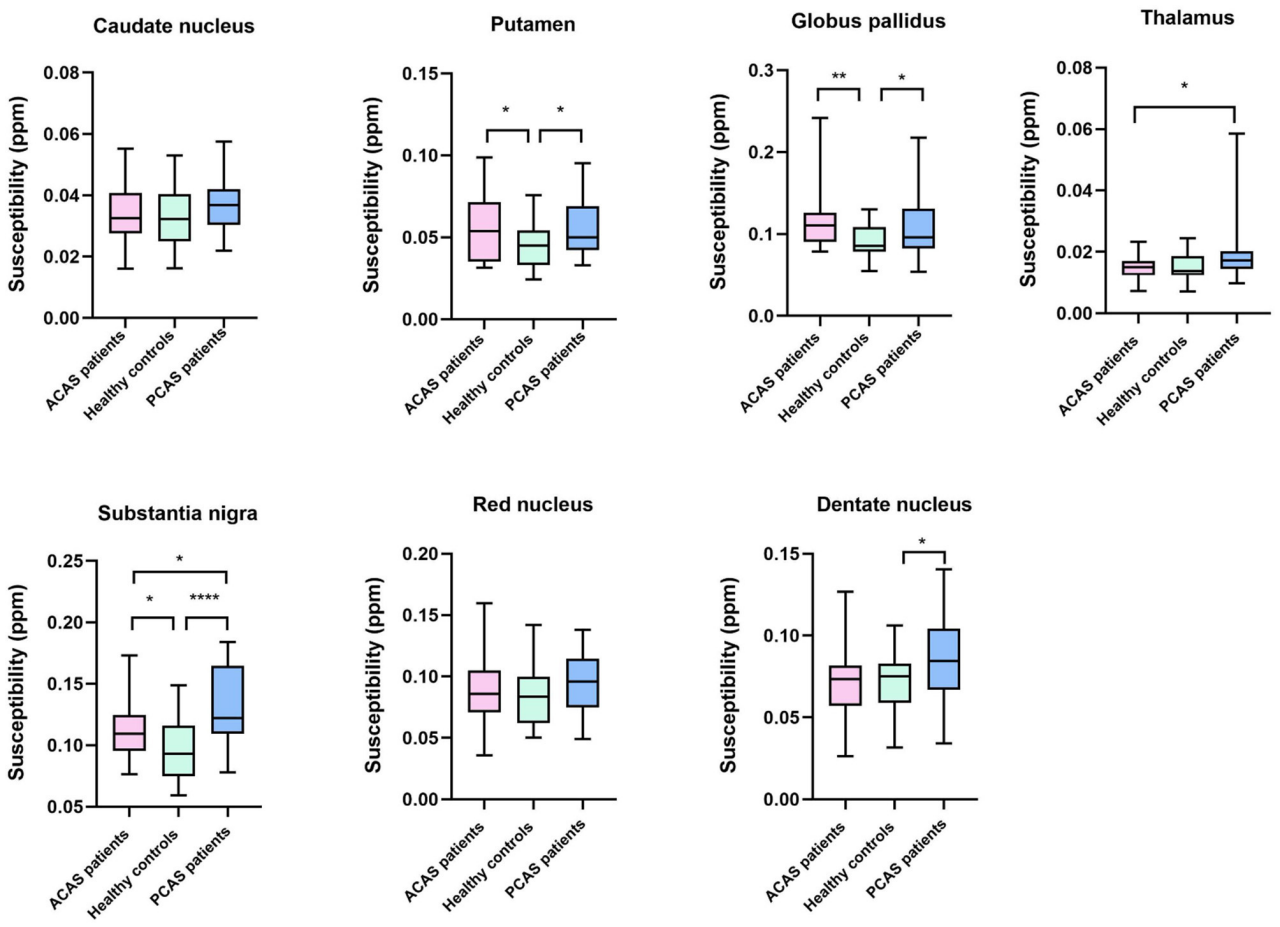
Box-and-whisker plots were used to compare the susceptibility of seven gray matter nucleus subregions across healthy controls, anterior circulation artery stenosis (ACAS) patients, and posterior circulation artery stenosis (PCAS) patients. Significant difference: *P < 0.05; **P < 0.01; ****P < 0.0001.

Using independent sample *t*-test, ACAS patients exhibited significantly higher susceptibility than healthy controls in bilateral PU, GP, and SN (mean: 0.0571 ± 0.0210 vs. 0.0454 ± 0.0140 ppm for PU, *P* = 0.025; mean: 0.1134 ± 0.0331 vs. 0.0910 ± 0.0200 ppm for GP, *P* = 0.006; mean: 0.1134 ± 0.0222 vs. 0.0965 ± 0.0232 ppm for SN, *P* = 0.011), while comparable susceptibility was found in the CN, TH, RN, and DN between both groups (all *P* > 0.05) (Table 2; Figure 2).

**Table 2:** Susceptibility (ppm) comparisons in gray matter nuclei between healthy controls and patients with ACAS.

**ROI**	**Healthy**	**Patients**	***t-value***	***P-value***
	**controls**	**with ACAS**		
	**(*n* = 25)**	**(*n* = 25)**		
CN	0.0334 ± 0.0104	0.0342 ± 0.0102	−0.247	0.806
PU	0.0454 ± 0.0140	0.0571 ± 0.0210	−2.316	0.025
GP	0.0910 ± 0.0200	0.1134 ± 0.0331	−2.898	0.006
TH	0.0154 ± 0.0043	0.0151 ± 0.0038	0.324	0.747
SN	0.0965 ± 0.0232	0.1134 ± 0.0222	−2.639	0.011
RN	0.0850 ± 0.0255	0.0898 ± 0.0278	−0.630	0.531
DN	0.0716 ± 0.0183	0.0730 ± 0.0240	−0.220	0.827

*ACAS, anterior circulation artery stenosis; ROI, region of interest; CN, caudate nucleus; PU, putamen; GP, globus pallidus; TH, thalamus; SN, substantia nigra; RN, red nucleus; DN, dentate nucleus*.

Independent sample *t*-test also showed no significant differences of susceptibility in the CN, TH, and RN between healthy controls and patients with PCAS (all *P* > 0.05, Table 2). However, compared with healthy controls, patients with PCAS presented significantly higher susceptibility in the PU, GP, SN, and DN, respectively (mean: 0.0454 ± 0.0140 vs. 0.0560 ± 0.0170 ppm for PU, *P* = 0.020; mean: 0.0910 ± 0.0200 vs. 0.1076 ± 0.0352 ppm for GP, *P* = 0.046; mean: 0.0965 ± 0.0232 vs. 0.1320 ± 0.0326 ppm for SN, *P* < 0.0001; mean: 0.0716 ± 0.0183 vs. 0.0873 ± 0.0275 ppm for DN, *P* = 0.022; Table 3; Figure 2).

**Table 3:** Susceptibility (ppm) comparisons in gray matter nuclei between healthy controls and patients with PCAS.

**ROI**	**Healthy**	**Patients**	***t-value***	***P-value***
**ROI**	**controls**	**with PCAS**		
**ROI**	**(*n* = 25)**	**(*n* = 25)**		
CN	0.0334 ± 0.0104	0.0372 ± 0.0095	−1.344	0.185
PU	0.0454 ± 0.0140	0.0560 ± 0.0170	−2.404	0.020
GP	0.0910 ± 0.0200	0.1076 ± 0.0352	−2.044	0.046
TH	0.0154 ± 0.0043	0.0193 ± 0.0094	−1.858	0.069
SN	0.0965 ± 0.0232	0.1320 ± 0.0326	−4.443	<0.000
RN	0.0850 ± 0.0255	0.0935 ± 0.0244	−1.198	0.237
DN	0.0716 ± 0.0183	0.0873 ± 0.0275	−2.373	0.022

*PCAS, posterior circulation artery stenosis; ROI, region of interest; CN, caudate nucleus; PU, putamen; GP, globus pallidus; TH, thalamus; SN, substantia nigra; RN, red nucleus; DN, dentate nucleus*.

In addition, mean susceptibility of bilateral TH and SN in PCAS patients was significantly higher than in ACAS patients (mean: 0.0193 ± 0.0094 vs. 0.0151 ± 0.0038 ppm for TH, *P* = 0.043; mean: 0.1320 ± 0.0326 vs. 0.1134 ± 0.0222 ppm for SN, *P* = 0.022), while comparable susceptibility was separately found in the CN, PU, GP, RN, and DN between both patient groups (all *P* > 0.05) (Table 4; Figure 2).

**Table 4:** Susceptibility (ppm) comparisons in gray matter nuclei between patients with ACAS and PCAS.

**ROI**	**Patients**	**Patients**	***t-value***	***P-value***
	**with ACAS**	**with PCAS**		
	**(*n* = 25)**	**(*n* = 25)**		
CN	0.0342 ± 0.0102	0.0372 ± 0.0095	−1.104	0.275
PU	0.0571 ± 0.0210	0.0560 ± 0.0170	0.198	0.844
GP	0.1134 ± 0.0331	0.1076 ± 0.0352	0.603	0.549
TH	0.0151 ± 0.0038	0.0193 ± 0.0094	−2.076	0.043
SN	0.1134 ± 0.0222	0.1320 ± 0.0326	−2.362	0.022
RN	0.0898 ± 0.0278	0.0935 ± 0.0244	−0.500	0.620
DN	0.0730 ± 0.0240	0.0873 ± 0.0275	−1.967	0.055

*ACAS, anterior circulation artery stenosis; PCAS, posterior circulation artery stenosis; ROI, region of interest; CN, caudate nucleus; PU, putamen; GP, globus pallidus; TH, thalamus; SN, substantia nigra; RN, red nucleus; DN, dentate nucleus*.

### Correlation Analysis of QSM Susceptibility With NIHSS and mRS Scores

**Figure 3:**
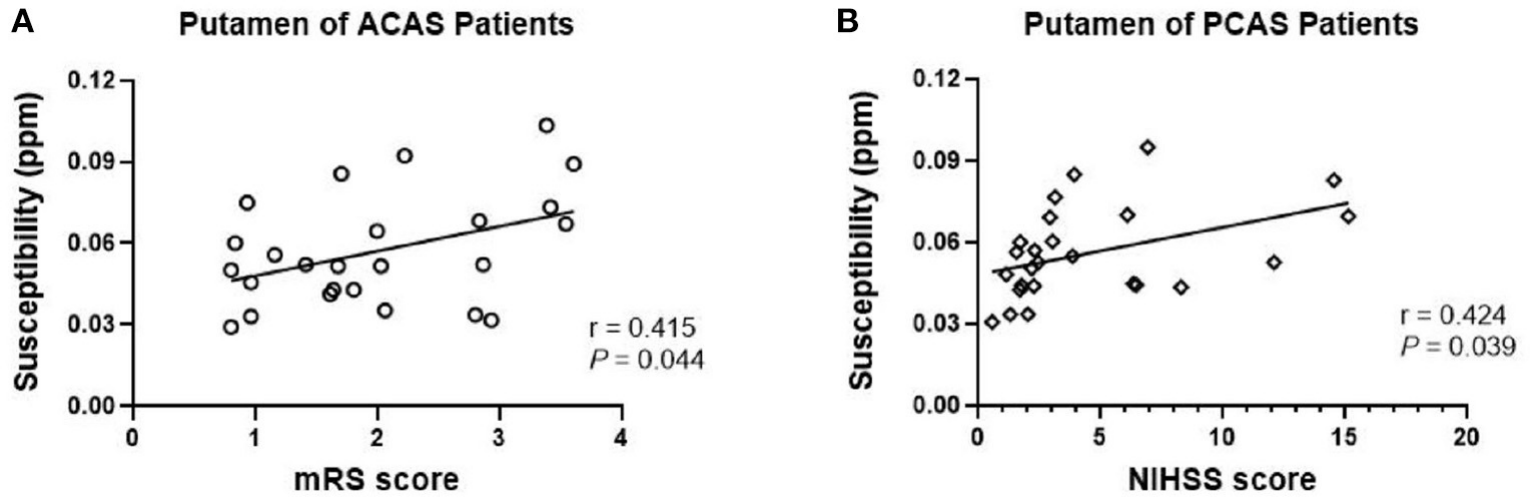
Partial correlation analysis between mean susceptibility at bilateral putamen (PU) with NIHSS and mRS scores, adjusted for age. **(A)** In anterior circulation artery stenosis (ACAS) patients, mean susceptibility of bilateral PU was significantly increased with mRS score (*r* = 0.415, *P* = 0.044); **(B)** In posterior circulation artery stenosis (PCAS) patients, mean susceptibility of bilateral PU was significantly increased with NIHSS score (*r* = 0.424, *P* = 0.039).

Partial correlation analysis, with age as a covariate, was further performed to separately evaluate the relationships of susceptibility levels at bilateral PU, GP, and SN in ACAS patient group and bilateral PU, GP, TH, SN, and DN in PCAS patient group with NIHSS and mRS scores. Susceptibility levels of bilateral PU in patients with ACAS were significantly increased with mRS score (*r* = 0.415, *P* = 0.044; Figure 3A). In addition, a significant positive correlation was shown between the average susceptibility of bilateral PU in PCAS patients and NIHSS score (*r* = 0.424, *P* = 0.039; Figure 3B).

## Discussion

In this study, we systematically investigated the changes of iron content in deep GM nuclei for patients with ischemic stroke secondary to chronic ACAS and PCAS by using QSM. Our results showed that, compared with healthy controls, mean susceptibility levels of bilateral PU, GP, and SN in patients with ACAS and of bilateral PU, GP, SN, and DN in patients with PCAS were significantly increased, indicating excess iron deposited in different subregions of deep GM nuclei after long-term ACAS and PCAS. In addition, mean susceptibility of bilateral TH and SN in PCAS patients was significantly higher than in ACAS patients, indicating different patterns of ICAS have different patterns of iron deposition. After adjustment for age, average susceptibility levels at bilateral PU of patients with ACAS were significantly correlated with mRS score, and at bilateral PU in patients with PCAS were correlated with NIHSS score.

QSM can be utilized to quantitatively analyze the susceptibility of bioactive metal, including iron and calcium, by using phase shift changes caused by magnetic susceptibility effect (28). In addition, QSM imaging avoids low-frequency phase shift artifacts at air-tissue interface due to the unwrapping of phase images and the removal of background fields, and thus, provides the susceptibility maps with high tissue contrast and spatial resolution (29). Consistently, previous research has confirmed that the susceptibility evaluated by QSM was positively correlated with chemically determined iron concentration in brain tissue, particularly in deep GM nuclei (16). Moreover, many studies indicated increased iron-related susceptibility in brain tissues, as assessed by QSM, in various neurological diseases, such as AD, PD, Huntington's Disease (HD), and amyotrophic lateral sclerosis (30–33). Therefore, it is reliable to apply non-invasive QSM imaging to investigate the changes of iron content in deep GM nuclei for patients with long-term ACAS and PCAS.

**Figure 4:**
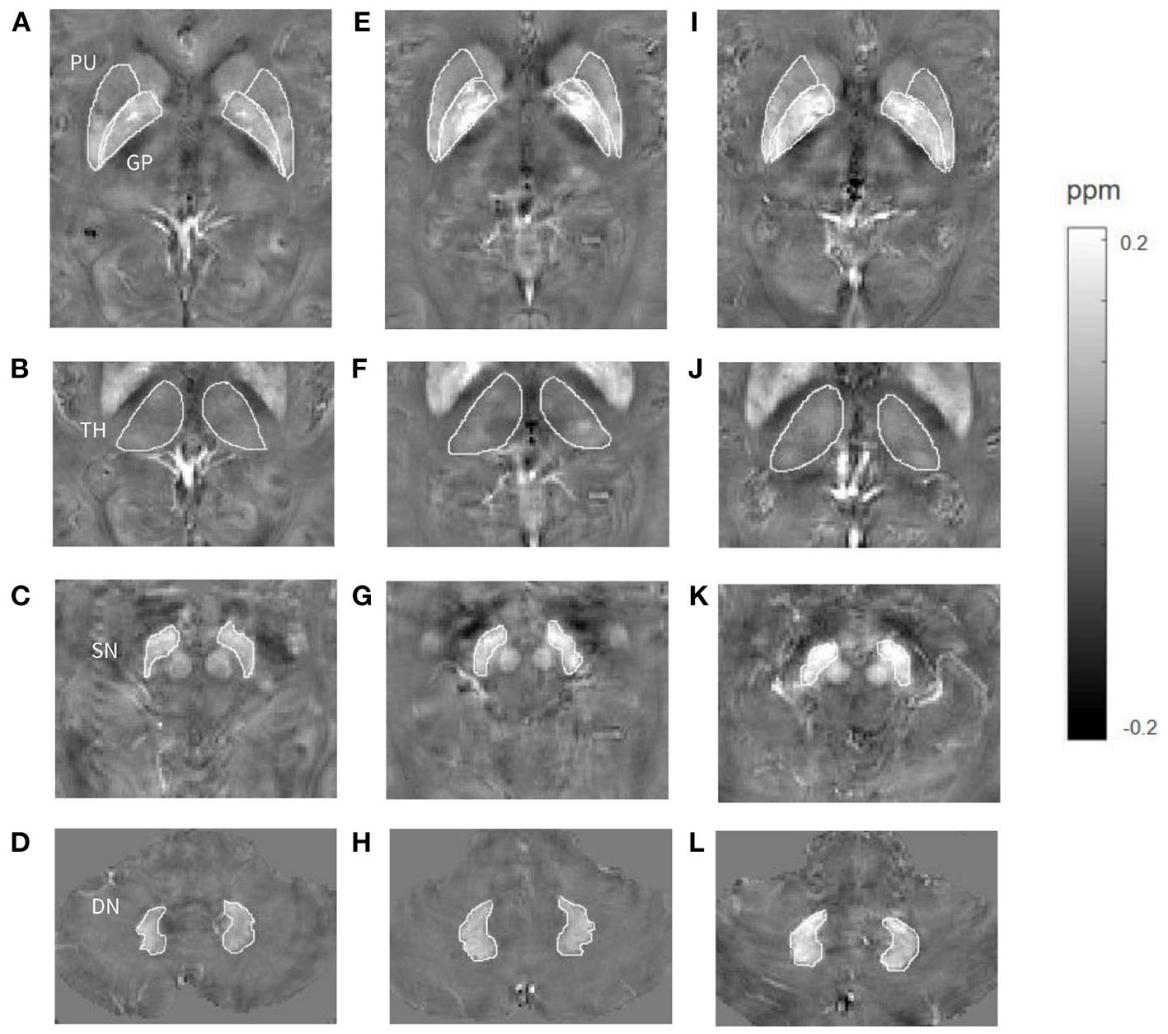
Comparisons of susceptibility in gray matter nuclei among a healthy control (**A–D**, a 65-year-old male), a patient with long-term anterior circulation artery stenosis (ACAS) (**E–H**, a 62-year-old male) and a patient with long-term posterior circulation artery stenosis (PCAS) (**I–L**, a 65-year-old male). Higher mean susceptibility of bilateral putamen (PU), globus pallidus (GP), and substantia nigra (SN) was shown in the ACAS patient, and of bilateral PU, GP, thalamus (TH), SN, and dentate nucleus (DN) was shown in the PCAS patient.

For patients with long-term ACAS, iron-related susceptibility of bilateral PU, GP, and SN was significantly higher than those in healthy controls (Figure 4), indicating that ACAS could result in cerebral iron metabolism disorders in some GM nucleus subregions. Similar to our finding, Du et al. (21) reported that average QSM susceptibility of bilateral PU in nine patients with MCA occlusion was significantly increased. However, increased iron-related susceptibility at bilateral GP of ACAS patients observed in our study was different from Du et al.'s study that lower susceptibility was revealed at bilateral GP in patients with MCA occlusion. In addition to larger sample size in our study, not identical patient types, of which Du et al. only focused on MCA occlusion, may also explain this discrepancy between two studies.

Increased iron-related susceptibility of bilateral PU, GP, SN, and DN was observed in patients with long-term PCAS relative to healthy controls (Figure 4). This result indicated that some GM nucleus subregions, including PU, GP, SN, and DN, were vulnerable to abnormal iron deposition after long-term PCAS. Liu et al. (34) used phase shifts from phase images of susceptibility weighted imaging to evaluate focal brain iron alterations in old patients with ischemic cerebrovascular disease, and found that iron content in PU and GP was significantly increased in patients with previous ischemic stroke history. This finding is consistent with ours. To be noted, our results demonstrated that mean susceptibility of bilateral SN and DN was significantly increased in patients with PCAS, which has not been reported previously. The basal ganglia and TH contain important structures closely associated with nervous system function. Therefore, these regions received highly clinical interest and were often investigated for ischemic stroke. Recently, in addition to basal ganglia and TH, alterations of iron content in other GM nucleus subregions, including SN, RN, and DN, have been confirmed to be closely related with many neurological disorders, such as PD, degenerative cerebellar ataxia, and CADASIL (24, 30, 35). Therefore, in this study, iron alterations of bilateral SN and DN in patients with PCAS and of bilateral SN in patients with ACAS may provide a new insight to understand the pathological mechanism of ischemic stroke caused by ICAS.

Interestingly, we found that for patients with ACAS, except for PU and GP regions where main blood supply comes from anterior circulation arteries, remote SN region also showed increased iron deposition. Similarly, in addition to SN and DN regions mainly supplied by posterior circulation arteries, the supratentorial PU and GP regions also presented elevated iron content in patients with PCAS. Previous studies have confirmed that after long-time cerebral ischemia and hypoxia, neuropathological changes not only occur in focal ischemia areas, but also in anatomically or functionally connected brain regions distant from ischemia areas (36, 37). In neuroanatomical and pathological studies, fibrous pathways between SN and striatum (CN, PU, and GP) have been extensively described (38). Neurodegeneration of SN secondary to ischemic injury of striatum has been widely reported in animal stroke models (39, 40). There may be an iron metabolism pathway of “cerebellum–brainstem (SN)–basal ganglia” in brain tissue, and iron is transported and metabolized in nerve cells along the above-mentioned nerve fiber pathway (41). When degeneration of nerve fibers occurs in any of the above parts or nerve conduction function is damaged by insufficient blood supply, iron cannot be excreted normally and excessive iron can deposit accordingly (41). Linck et al. (42) utilized R2^*^ mapping to explore iron alterations of SN in patients with supratentorial ischemic stroke, and found that SN ipsilateral to the lesion presented greater iron content 1 year after stroke. Indeed, our results of both ACAS and PCAS patients could indirectly demonstrate the existence of known fibrous pathways between SN and basal ganglia.

Compared with ACAS patients, increased susceptibility of bilateral TH and SN was observed in PCAS patients, indicating that specific GM nuclei subregions, including TH and SN, were more vulnerable to abnormal iron deposition after long-term PCAS than ACAS (Figure 4). It is well-known that the main blood supply of TH and SN comes from posterior circulation arteries, so both regions may be more susceptible to increased iron deposition after PCAS. Interestingly, there was no different susceptibility in bilateral TH between healthy controls and patients with PCAS, but the susceptibility of TH in PCAS patients showed a higher trend toward healthy controls with *P*-value of 0.069. We speculate that, given a larger sample, TH may exhibit significantly increased susceptibility in PCAS patients than in healthy controls. Similar to our study, in a previous study that used signal intensity variation on T2^*^-weighted images to reflect the changes of iron content, ischemic stroke patients, including those with infarcts in the distribution of PCA, showed increased iron accumulation in TH on the side ipsilateral to the infarct (43). Compared with healthy controls, both ACAS and PCAS patients exhibited significantly increased susceptibility in PU and GP. However, no significant difference of susceptibility was revealed in PU and GP between both patient cohorts. This may be because that the effect of ACAS on iron deposition in PU and GP regions, mainly supplied by anterior circulation arteries, is weaker than that of PCAS on SN regions supplied dominantly by posterior circulation arteries. As mentioned above, there may be an iron metabolism pathway between SN and basal ganglia (41). Therefore, iron deposition in SN was significantly affected by PCAS, resulting in increased iron deposition in PU and GP for PCAS patients. That might explain why comparable susceptibility was found in the PU and GP between both patient groups. From the comparison results of both patient cohorts, it suggested that specific GM nuclei subregions, including TH and SN, were more vulnerable to abnormal iron deposition after PCAS than after ACAS. Currently, few studies have focused on iron deposition in GM nuclei after PCAS, so further studies with larger samples are needed to explore different iron deposition patterns after ACAS and PCAS.

In addition, our study showed that after adjustment for age, average susceptibility levels of bilateral PU in patients with ACAS were positively correlated with mRS score, and of bilateral PU in patients with PCAS were positively correlated with NIHSS score. NIHSS and mRS scores are the most commonly used clinical scales to evaluate the degree of neural function deficiency in ischemic stroke. Higher NIHSS or mRS scores indicate more severe neurological deficit. Therefore, for both ACAS and PCAS patients, increased iron accumulation in PU was found associated with worse neural function in our study. PU is an important component of basal ganglia, playing an important role on normal brain function and behavior. A previous study has shown that PU abnormalities found by resting-state functional MRI were associated with clinical pain and motor disturbance in complex regional pain syndrome (44). Domínguez et al. (45) found that iron related QSM susceptibility of PU in HD patients was higher than those in healthy controls, and iron content of PU in symptomatic HD patients was positively correlated with disease severity. Another study suggested that iron content of the left PU was significantly increased in type 2 diabetes with mild cognitive impairment and it was closely correlated with cognitive deficits (46). Our findings also supported that increased iron accumulation in PU was closely associated with neural function deficiency in patients with ACAS and PCAS. However, we are not able to illustrate whether neurological dysfunction after ischemic stroke leads to increased iron deposition or iron deposition exacerbates neurological deficits in the current study. Therefore, long-term follow-up research in evaluating changes of iron levels and neurological deficits with a large clinical cohort is requested to further investigate this aspect.

In ischemic stroke, the physiological and pathological mechanisms of neuronal damage caused by long-term ischemia and hypoxia are complex, involving free radical damage, inflammatory overactivation, toxic effects of excitatory amino acids, apoptosis or necrosis, etc. (47). Among them, iron-mediated free radicals play an important role in ischemic brain injury. Iron is a catalyst of biomolecular oxidative damage and an important factor of oxidative stress. Iron overload enhances Fenton reactions to produce a large number of toxic hydroxyl radicals, and these free radicals attack the unsaturated lipid chains on the cell membrane to cause lipid peroxidation (48). Lipid peroxidation not only occurs immediately after cerebral ischemia/reperfusion, but also continues to promote neuronal death for a long time after ischemia/reperfusion (49). In addition to catalyzing the production of free radicals and promoting lipid peroxidation, iron also has a direct toxic effect. Excessive iron can combine with intracellular organic compounds to produce highly reactive iron or ferric ions, causing damage to lysosomal, and mitochondrial membranes and further leading to degeneration of cell structure and function (50). Therefore, in this study, increased iron deposition in specific GM nucleus subregions observed in ACAS and PCAS patients may contribute to neuronal damage and provide a possible explanation for the neurological symptoms after ischemic stroke.

This study has several limitations. First, a limited sample size was applied in this study, which could introduce potential selection bias. Second, approximate degree of neurological impairment in patients with ACAS and PCAS was evaluated using NIHSS and mRS scores, but extensive clinical evaluations, including motor, language and cognitive functions, were not assessed in this study. Third, as a cross-sectional study, we could not observe the dynamic relationship between neurological deficit and iron content in specific GM nucleus regions. Therefore, follow-up longitudinal studies in a larger clinical cohort with extensive clinical evaluations to look at people with ICAS and monitor their iron deposition and degree of neurological impairment are requested for further validation.

## Conclusion

In conclusion, using QSM imaging, several GM nucleus subregions, including bilateral PU, GP, and SN in ACAS patients and PU, GP, SN, and DN in PCAS patients, exhibited increased iron deposition, indicating that abnormal iron metabolism may present in different subregions of deep GM nuclei after long-term ACAS and PCAS. In addition, iron levels of bilateral TH and SN in PCAS patients were significantly higher than in ACAS patients, indicating that specific GM nuclei subregions were more vulnerable to abnormal iron deposition after PCAS than after ACAS. Iron content of PU in patients with ACAS and PCAS was correlated with neurological deficit scores. Therefore, iron quantification measured by QSM susceptibility may provide a new insight to understand the pathological mechanism of ischemic stroke caused by ACAS and PCAS, and iron deposition may play an important role in the process of neurological deficits in ischemic stroke.

## Data Availability Statement

The original contributions presented in the study are included in the article/Supplementary Material, further inquiries can be directed to the corresponding author.

## Ethics Statement

The studies involving human participants were reviewed and approved by Medical Ethics Committee of First Affiliated Hospital of Shandong First Medical University. The patients/participants provided their written informed consent to participate in this study. Written informed consent was obtained from the individual(s) for the publication of any potentially identifiable images or data included in this article.

## Author Contributions

HM, WD, and XW designed this study, interpreted the imaging data, making statistical analysis, and wrote the manuscript. KC, XW, YG, and CZ collected the clinical data, did the MRI scanning, and revised the manuscript. All authors contributed to the article and approved the submitted version.

## Funding

This study was supported by Shandong Science and Technology Development Plan Project 2009GG10002024.

## Conflict of Interest

WD was employed by company GE Healthcare. The remaining authors declare that the research was conducted in the absence of any commercial or financial relationships that could be construed as a potential conflict of interest.

## Publisher's Note

All claims expressed in this article are solely those of the authors and do not necessarily represent those of their affiliated organizations, or those of the publisher, the editors and the reviewers. Any product that may be evaluated in this article, or claim that may be made by its manufacturer, is not guaranteed or endorsed by the publisher.
