# Supplementary material for: Iron Deposition in Gray Matter Nuclei of Patients With Intracranial Artery Stenosis: A Quantitative Susceptibility Mapping Study
Source: Front Neurol. 2022 Jan 5;12:785822. doi: 10.3389/fneur.2021.785822 (PMC8766754; doi:10.3389/fneur.2021.785822)
Supplement: Supplementary file 1 [file Table_1.DOC]

**Supplementary Tables**

| **Supplementary Table 1. ICC of agreement between two radiologists for susceptibility measurements** | | |
| --- | --- | --- |
| ROI | ICC of left cerebral hemisphere (95% CI) | ICC of right cerebral hemisphere (95% CI) |
| CN | 0.858 (0.785 to 0.908) | 0.860 (0.787 to 0.909) |
| PU | 0.937 (0.902 to 0.960) | 0.959 (0.935 to 0.974) |
| GP | 0.932 (0.895 to 0.956) | 0.960 (0.938 to 0.975) |
| TH | 0.890 (0.831 to 0.929) | 0.850 (0.772 to 0.902) |
| SN | 0.958 (0.935 to 0.973) | 0.938 (0.903 to 0.960) |
| RN | 0.917 (0.871 to 0.947) | 0.929 (0.889 to 0.954) |
| DN | 0.957 (0.930 to 0.973) | 0.953 (0.927 to 0.970) |
| ICC, intra-class correlation coefficient; ROI, region of interest; CI, confidence interval; CN, caudate nucleus; PU, putamen; GP, globus pallidus; TH, thalamus; SN, substantia nigra; RN, red nucleus; DN, dentate nucleus. | | |

| **Supplementary Table 2. Susceptibility (ppm) comparisons between bilateral gray matter nuclei**  **in healthy controls, patients with ACAS, and patients with PCAS** | | | | | | | | | | | | |
| --- | --- | --- | --- | --- | --- | --- | --- | --- | --- | --- | --- | --- |
| ROI | Healthy controls  (n = 25) | | *t value* | *P value* | Patients with ACAS  (n = 25) | | *t value* | *P value* | Patients with PCAS  (n = 25) | | *t value* | *P value* |
|  | Left | Right |  |  | Left | Right |  |  | Left | Right |  |  |
| CN | 0.0341 ± 0.0117 | 0.0328 ± 0.0100 | -1.076 | 0.292 | 0.0347 ± 0.0108 | 0.0336 ± 0.0115 | -0.566 | 0.577 | 0.0376 ± 0.0113 | 0.0369 ± 0.0123 | -0.235 | 0.816 |
| PU | 0.0454 ± 0.0136 | 0.0454 ± 0.0149 | -0.066 | 0.948 | 0.0555 ± 0.0213 | 0.0587 ± 0.0245 | 0.854 | 0.402 | 0.0558 ± 0.0154 | 0.0562 ± 0.0206 | 0.126 | 0.901 |
| GP | 0.0903 ± 0.0211 | 0.0917 ± 0.0200 | 0.730 | 0.472 | 0.1095 ± 0.0266 | 0.1174 ± 0.0427 | 1.495 | 0.148 | 0.1067 ± 0.0364 | 0.1085 ± 0.0361 | 0.499 | 0.622 |
| TH | 0.0150 ± 0.0044 | 0.0159 ± 0.0045 | 1.709 | 0.100 | 0.0142 ± 0.0057 | 0.0159 ± 0.0045 | 1.223 | 0.233 | 0.0201 ± 0.0110 | 0.0184 ± 0.0090 | -1.186 | 0.247 |
| SN | 0.0956 ± 0.0223 | 0.0973 ± 0.0259 | 0.591 | 0.560 | 0.1107 ± 0.0247 | 0.1161 ± 0.0231 | 1.531 | 0.139 | 0.1316 ± 0.0350 | 0.1324 ± 0.0321 | 0.244 | 0.810 |
| RN | 0.0856 ± 0.0266 | 0.0845 ± 0.0255 | -0.504 | 0.619 | 0.0900 ± 0.0276 | 0.0896 ± 0.0294 | -0.135 | 0.894 | 0.0943 ± 0.0231 | 0.0926 ± 0.0275 | -0.577 | 0.569 |
| DN | 0.0725 ± 0.0178 | 0.0707 ± 0.0203 | -0.859 | 0.399 | 0.0744 ± 0.0249 | 0.0715 ± 0.0241 | -1.386 | 0.178 | 0.0880 ± 0.0303 | 0.0866 ± 0.0251 | -0.768 | 0.450 |
| ACAS, anterior circulation artery stenosis; PCAS, posterior circulation artery stenosis; ROI, region of interest; CN, caudate nucleus; PU, putamen; GP, globus pallidus; TH, thalamus; SN, substantia nigra; RN, red nucleus; DN, dentate nucleus. | | | | | | | | | | | | |
